# Laws of Nature

**Published:** 1902-08

**Authors:** E. G. Ferguson

**Affiliations:** Macon, Ga.


### LAWS OF NATURE.

BY E. G. FERGUSON, M.D.,
MACON, GA.

I shall not attempt to satiate literary appetites of cognoscent hearers with an intellectual feast on profound matters of medical science; neither shall I horrify them by viewing through a powerful lens the bacteria shooting forth in their fevered systems, nor shall I follow the wrigglings of the trichina spiralis to be found in the back of the porcine race. I do not propose to employ vague medical nomenclature or phraseology so as to obscure my ignorance or mystify my hearers into accepting my views, but try to convince you by appealing to your common sense by telling you in as plain and unvarnished a manner as my ability will admit a series of facts gathered from observing nature's laws, and exert my utmost efforts to show you the direct cause of nearly all the diseases man is heir to, and the simplicity of rules to be adopted for their avoidance. Many condemn without reflection new ideas advanced because they do not emanate from acknowledged teachers, or because they are at variance with accustomed dogmas, while the most complete absurdities uttered by authors of acknowledged repute are unhesitatingly gulped down for gospel truths. We are too prone to blindly follow in the footsteps of our predecessors and take for granted the sum of their observations without exercising our own faculties to question the correctness of their views. We are slaves to custom, habit and fashion. Years of bitter experience will only suffice to change our views and cause us to abandon absurdities clung to from fashion or habit, even though felt to be fallacious and injurious. Within the recollection of many present it was thought by leading lights in the profession that to drink cold water in fever was certain death. Nature's efforts to effect a cure was received with unfeigned horror, and the suffering patient, imploring heaven to soften the hearts of his loving friends that they might allow him a free drink of cold water, was enjoined by his relatives to follow the advice of the doctor, as he was an impartial judge and knew what was best; that he (the patient), under fever, was not a rational judge and ought to be restrained from such madness as drinking cold water. Had they reflected for a moment upon the comfort of cold water to a fevered surface of inflammation, or the stinging heat occasioned by a burn, could they not readily have seen that a fire raging in the tissues was creating the desire expressed by nature in the call for that most comforting of all beverages to the fever-stricken, a glass of ice-water. How many instances have come under the observation of every member of this society where, unawares to the nurse and in the face of the injunction of the doctor, the patient has seized an opportunity in their absence to drink freely of cold water, completely revolutionizing the symptoms, of which the doctor took the credit to his skill, and not until after months of blissful ignorance did his patient inform him of his error? How many hundreds and thousands have been murdered by loving hands through the ignorant arrogance of limbs of the medical profession dictating to God by restraining those loving hands from complying with nature's laws in administering a draught of cold water? Had these would-be guides or correctors of nature's supposed deficiencies a thorough knowledge of the elementary constituents of the human body, they would be aware of the fact that, in an average-sized human being, between one-third and one-fourth is solid matter, the other two-thirds or three-fourths being liquid, and that water constitutes this three-fourths, and would thus administer water. The economy of the human body is one of constant supply and waste. The stomach receives the food, and in that organ it comes in contact with the solvent fluid, the gastric juice, where it is partially dissolved. It is then propelled into the duodenum, where it comes in contact with the juices of the pancreas for further digestion, and in contact with the bile to prevent fermentation. Passing down the entire length of the intestinal canal, at various points this liquid, or portions of it, is absorbed and carried to all parts of the body through the lacteals and blood-vessels to replenish waste tissue. The stomach is intimately connected through the great sympathetic nervous system with all parts of the body, but more particularly and directly with the brain through the pneumogastric, as is illustrated in a case of concussion of the brain, if sufficient time has elapsed or reaction sets in, when the stomach revolts and vomiting ensues, although no direct injury is inflicted upon the stomach. Again, injury inflicted upon the stomach will show in the brain a headache, or, if severe enough, coma.

I have witnessed cases of coma produced by excessive indulgence in food, and so soon as emesis could be induced the patient was fully restored to consciousness, but with a severe headache. During this period of coma respiration was deep and sighing, extremities cold, pupils dilated and pulse slow and compressible.

Since we have seen such astounding facts in connection between the stomach and brain and all parts of the body, is it not reasonable to suppose that this is the great channel through which nearly all our ills begin? Assuming that it is, what ought we to do to obviate these troubles? Simply observe nature's laws. Never eat until hungry nor drink until thirsty, and rest when tired. The nice point for the preservation of health for one to know is when sufficient food has been taken and the proper sort to take. Every man, woman and child ought to be the proper judges of this for themselves, but I am sorry to say that human beings display less sense than the brute creation. Feed a dog all he can eat, and after his appetite is fully satiated he refuses to indulge further. Not so, however, with this greatest of God's handiwork, man. He must eat because he can, stuff his stomach to an uncomfortable distention, and then acknowledge he has eaten too much. After a sufficient amount of nourishment has been taken a feeling of warmth and comfort is experienced, with perhaps an inclination to rest, which, after a period, is succeeded by feelings of renewed vigor—that is, provided the food is digestible, and not too much in quantity, for quantity is as injurious as quality. It ought to be thoroughly masticated and never eaten hurriedly. If eaten hurriedly one is liable to eat to excess. How often do we find mothers feeding toothless infants with such food as they themselves partake of, and when a case of cholera infantum is set up the mother shields herself generally, with the aid of the physician, behind the delusive subterfuge that the child is teething. No fault of the mother that the disease was brought on, oh no! In the face of a law written as plainly as the toothless mouth of her babe can convey it to her, she sets aside the decree and bids defiance to the consequences, and fills the little stomach, that was never intended for other than the nourishment her breast affords, with such articles as she in her crude judgment thinks it ought to have. If God Almighty intended such food was requisite for the sustenance of that child he would have ordained that it should be born with a full set of teeth. There is not a single member of the profession here but can bear testimony to the truth of my assertion, that, after having partaken too freely of the luxuries of the table, a feeling of indolence, followed by a strong desire for rest, is the immediate sensation experienced from this indiscretion. Does this not show that we ought to indulge nature's demand instead of hurrying off to engage in business pursuits? Examples are numerous in the brute creation. As soon as the cow, hog, dog or horse has fully satiated its appetite rest is sought and the feeling gratified, for we see each complying with that demand. Indigestible food is productive of the same feeling, intensified to such a degree as to cause the whole system to sympathize and manifest that sympathy in various revolutions. Would-be kind friends, visiting those afflicted with fever, advise a little stimulant or nourishing food, as a good beefsteak, soft-boiled eggs, a light battercake fried in grease, soda-crackers, milk, toast and tea, and all sorts of indigestible matter, at the thought of which the fevered stomach revolts, and, on attempting to introduce into that organ, are peremptorily refused admission, vomiting ensues, to the great distress of the patient but to the alleviation of the disease, for the very sick stomach is the loving watchdog of this distressed and fevered system, preventing further trifling with nature while she is attempting to correct that disordered system brought on by violating her laws. This sick stomach says more plainly than words that the system does not require fresh replenishment; that it has trouble now from that very source, dating weeks and months back, and asks that nature be first permitted to free herself of the superabundance already there before taking more.

Another flagrant violation of nature's laws, and I verily believe the chief source from which we draw all our ills, and yet the one most urged upon by the great majority of our practitioners, is regularity of habits of life. Be regular to eat, be regular to sleep, etc., are the commands of nine-tenths of the practitioners of the present day, as though the system was a piece of machinery like a locomotive, capable of being handled at will, or like the clock that ticks behind the door, needs to be wound up regularly to run a given length of time. Load the stomach three times a day, whether it calls for food or not. Drink all sorts of slops out of all season, whether he thirst or not. Force nature to do as we think best. Don't consult her on any topic, but bend her laws to suit our will. Verily, he who would set himself up as her adviser is the god of God. The process of waste and decay does not go on with the regularity of the click of a clock, nor is it within the pale of human power to control, but goes on in smooth compliance with nature's laws until combustion exhausts the vital force and gradually stops the machine. The law, like the handwriting on the wall, points to the inevitable end. If we comply with that law, all goes on smoothly and well, and if we refuse nature's counsel her decree is uttered sooner or later. The stomach calls for food through the agency of the appetite just as soon as the system requires it, and no sooner; it asks for drink just as soon as the system needs liquid, and no sooner. The whole economy calls for rest just as soon as it needs rest, and no sooner; yet we pigmies of God's great handiwork set up a dictatorship each for himself, and generally before the expiration of the time alloted for his existence kicks himself out of office and out of the world.

The fundamental cause, then, of all our ills arises from the stomach. In youth absorption goes on more rapidly than excretion, or endosmosis takes place more rapidly than exosmosis, for if this was not the case we never would grow in stature or weight, but remain in abeyance as the midgets or other eccentricities in nature, resulting from an abnormal condition of those laws whereby excretion is equal, or almost equal, to absorption. These laws of absorption and excretion maintain equipoise of health, and any deviation from the balance is manifest in disturbances of other functions. Food in excess of the requirements of the system may be absorbed without a corresponding elimination and obesity result, and *vice versa*, or a lack of the requisite amount of nourishment causes emaciation. When the excretories are tardy in their functions accumulation of effete matter occurs; then nature makes an effort to rid herself of this foreign matter that is no longer of use, and we have fever or combustion in excess to consume waste tissues, tissues that from undue feeding accumulate in all parts of the body and obstruct the even functions of the machinery of the system; or if not directly in fever we have it in other forms in local inflammations, as in acute rheumatism, neuralgia, etc., where it has poisoned the nervous system.

Since such dire effects are coincident with excess of food, may there not be articles less suitable for the replenishment of the system, and others more conducive to health? And how distinguish them? I do not claim to be a Huxley, Darwin, or Herbert Spencer, or to emulate the doctrines set forth by these able scholars of science, whose reasonings have never been refuted; but on the contrary am becoming more firmly fixed in my own insignificance that stimulates me to avoid the beaten track and look for further truths. I do claim that calm, unprejudiced reason and careful comparison of the human race with other animals will teach us lessons of incalculable value in enabling us to live naturally. In comparing the human structure with the higher orders of the lower animal creation, we are forced to admit the homology that so clearly exists, and when we come to look at nature untrammeled by the bloodhounds of civilization, unrestrained in the pursuit of health and pleasure by the customs of society, we feel our insignificance the more keenly, as we pride ourselves on being the only animal endowed with reasoning powers. How many female wrecks flit before your mind's eye, hurrying to an untimely end, digging their own graves and absolutely signing their own death-warrants as they buckle on their corsets? Upon a careful examination of the habits of those higher orders of the lower animal creation, as the Quadrumana, we find these animals subsisting almost exclusively, with but few exceptions, on fruits, nuts and plants (some few eat insects and bird eggs), and amongst the anthropomorphous apes we find some of these species even make shelter of bark gathered from rotten logs amongst the branches of trees. Examine the mouths of these animals and see what a close similarity in shape and arrangement of teeth they bear to ours. Again, compare the Carnivora and man, and we find but an analogue of the most distinguishing characteristic of the Carnivora in the canine tooth of the human, showing most unerringly that we are not adapted to the indulgence of the excess of animal food we so habitually stuff ourselves with. That the article is truly pernicious to the human economy has daily been admitted. Beef essences, animal broths and soups so highly lauded in former times in wasting diseases are now pretty generally being abandoned for the more assimilable farinaceous articles so abundantly in nature and readily prepared. That the ruminants now indulged in as food are positively detrimental to the human system was long since proven by Banting of England, who reduced himself from an object of disgusting obesity to the size of an ordinary man by the rigid and undeviating indulgence in animal food to the total exclusion of all other articles. Never allow anything to enter the mouth that is offensive to the nose; never swallow anything offensive to the taste. These are the watch-dogs of the stomach. Never eat until hungry, and you will never be sick.

